# Vitamin D receptor activation reduces inflammatory cytokines and plasma MicroRNAs in moderate chronic kidney disease – a randomized trial

**DOI:** 10.1186/s12882-017-0576-8

**Published:** 2017-05-16

**Authors:** Ladan Mansouri, Kristina Lundwall, Ali Moshfegh, Stefan H. Jacobson, Joachim Lundahl, Jonas Spaak

**Affiliations:** 1 Immunology and Allergy Unit, Department of Medicine Solna, Karolinska Institutet, Karolinska University Hospital, Stockholm, Sweden; 2 Department of Clinical Sciences, Karolinska Institutet, Danderyd University Hospital, Stockholm, Sweden; 3 Department of Oncology-Pathology, Karolinska Institutet, Karolinska University Hospital, Stockholm, Sweden

**Keywords:** Chronic kidney disease, Endothelial function, Inflammation, VEGF, Micro RNA, Paricalcitol

## Abstract

**Background:**

Chronic kidney disease (CKD) is a major risk factor for cardiovascular disease (CVD), partly due to endothelial dysfunction and chronic inflammation**.** Vitamin D treatment in end stage renal disease is suggested to modulate the immune system and lead to improved outcomes. We and others have demonstrated that treatment with vitamin D or activated vitamin D analogues protects the endothelial function in less severe renal disease as well. Since the endothelial protection might be mediated by vitamin D effects on inflammation, we assessed levels of pro-inflammatory cytokines and micro RNAs (miRs) in patients with moderate CKD, treated with an active vitamin D analogue (paricalcitol).

**Methods:**

Thirty-six patients with moderate CKD were randomized to 12 weeks treatment with placebo, 1 μg, or 2 μg paricalcitol daily. Cytokines were measured by Milliplex 26-plex. Total RNA was isolated from plasma and miRs were determined by quantitative reverse transcription PCR analysis.

**Results:**

Selected pro-inflammatory cytokines decreased significantly following treatment, while no change was observed in the placebo group. The micro RNAs; miR 432-5p, miR 495-3p, and miR 576-5p were significantly downregulated in the active treated groups, compared to the placebo group.

**Conclusion:**

Paricalcitol treatment for 12 weeks in patients with moderate CKD reduces cytokines and micro RNAs involved in atherosclerosis and inflammation. The potentially protective role of vitamin D receptor activation in the inflammatory processes regarding the long-term outcomes in CKD patients warrants further studies.

**Trial registration:**

SOLID study; NCT01204528, April 27, 2010.

## Background

Chronic kidney disease (CKD) and microalbuminuria affect 10–13% of the population, and continue to increase in prevalence. In recent years, CKD has emerged as one of the strongest risk factors for cardiovascular diseases (CVD), in parity with diabetes mellitus, hypertension, smoking and hyperlipidemia [1]. Patients with CKD also have a worse prognosis after cardiovascular events [2]. This is at least in part due to vascular calcification, endothelial dysfunction, chronic inflammation, activated sympathetic nervous system and activated renin-angiotensin-aldosterone system (RAAS) [1–3].

Endothelial dysfunction is evident in all stages of CKD, and inflammation is a key factor in this process [2, 4]. Both the innate and the adaptive immune systems are dysregulated in CKD, and patients display increased levels of cytokines and acute phase proteins, as well as dysfunctional immune cells [5–7]. Cytokines and chemokines are important in the functionality of immune cells through the cellular signaling pathways and it is likely that cytokines are not only markers but also mediators in kidney disease and CVD [8].

Additionally, in recent years, genetic regulation has gained attention as a contributor to the process. Epigenetic modifications can alter gene expression, which ultimately appear as a phenotype. Micro RNAs (miRs) regulate gene expression by binding to mRNAs, resulting in their silencing or degradation [9]. Several miRs are involved in normal kidney function and some seem to contribute to the disease process [10–12].

Vitamin D deficiency is correlated to increased risk of cardiovascular events [13], and since CKD patients have low levels of active vitamin D, supplementation or treatment with activated vitamin D or analogues is considered a promising therapy. Treatment and/ or supplementation may reduce albuminuria, and cardiovascular events in patients with end stage renal disease (ESRD) [14, 15]. Four recently published interventional studies support the hypothesis that this protective effect is mediated, at least in part, by an improved endothelial function [16–19]. Moreover, the active form of vitamin D can modulate the functionality of immune cells by regulating the expression of a range of genes involved in the immune system [20, 21].

In the randomized SOLID trial [17], we recently demonstrated that treatment with a vitamin D receptor activator (VDRA)- paricalcitol, in patients with moderate CKD was associated with preserved micro- and macro-vascular endothelial function and microcirculation. In this study, we therefore hypothesized that this effect is a consequence of changes in pro-inflammatory cytokines, and altered up-stream gene expression regulators, assessed by plasma miRs.

## Methods

### Study design and procedures

This was a post-hoc sub-study of all subjects included in the SOLID-trial. The study design, and full report of the examinations has already been presented [17]. In brief, 36 patients were recruited in three groups; placebo, 1 μg paricalcitol, or 2 μg paricalcitol daily, in a randomized, double-blinded manner. To avoid the influence of sun exposure, no patients were included during June–August. The study started with two weeks of placebo, followed by 12 weeks of intervention. Blood samples were drawn at baseline and after 12 weeks, in the morning, after 12 h fasting and 20 min rest. All examinations were performed in research laboratories at Danderyd University Hospital and Karolinska Institutet, Stockholm, Sweden.

### Study population

Between June 2010 and February 2013, 36 patients were recruited from the Department of Nephrology at Danderyd University Hospital, Stockholm, Sweden. Patients were considered eligible if they had an estimated glomerular filtration rate (eGFR) of 15–59 mL/min/1.73 m^2^ calculated from plasma creatinine, using the MDRD formula, age > 20 years, a calcium level below 2.6 mmol/L, a plasma PTH level of 3,7–53 pmol/mL, a serum albumin above 30 g/L and had stable blood pressure < 150/100 mmHg with no change in angiotensin converting enzyme inhibitor (ACE-I) or angiotensin receptor blocker (ARB) medication, during two months before enrolling in the trial. Exclusion criteria were diabetes mellitus, ongoing treatment with vitamin D or its analogues, treatment with high doses of steroids or immune suppressants, nephrotic syndrome, renal artery stenosis, severe kidney stones, or severe disease such as active cancer, HIV or end stage congestive heart failure, acute renal failure during the last three months, and need for dialysis within six months. They were also excluded if they had uncontrolled hypertension (repeated measures of a brachial blood pressure > 150/100 mmHg). Table 1 presents the demographic characteristics of study participants at baseline. The regional Ethics Committee of Stockholm, Sweden, approved the study protocol and all patients provided written informed consent. The trial was registered on clinicaltrials.gov (SOLID study; NCT01204528).

**Table 1:** Demographic characteristics of participants at baseline

	Placebo (*n* = 12)	Paricalcitol 1 μg (*n* = 12)	Paricalcitol 2 μg (*n* = 12)
Age (y), mean (SD)	70.8 (10.0)	66.1 (7.9)	59.1 (11.6)
Sex (n, % male)	9 (75%)	11 (92%)	8 (67%)
eGFR^a^ (mL/min/1.73 m2), mean (SD)	42.1 (8.0)	38.9 (13.6)	40.6 (12.7)
Smoker (n)	1	1	0
Hypertension (n)	3	4	4
History of myocardial infarction (n)	3	2	0
History of atrial fibrillation (n)	1	1	0
History of stroke (n)	3	0	0
History of transient ischaemic attack (n)	1	0	0
History of heart failure (n)	0	0	1
History of aortic aneurysm (n)	0	1	1
CKD duration (y), mean (SD)	10.3 (8.8)	5.8 (6.0)	9.7 (10.5)
ACE-I^b^/ ARB^c^ (n)	11	9	9
β-blockers (n)	6	8	4
Calcium channel blockers (n)	10	8	4

^a^Estimated glomerular filtration rate

^b^Angiotensin converting enzyme inhibitor

^c^Angiotensin II receptor blocker

### Characterization of pro-inflammatory cytokines and chemokines in plasma

The concentration of immune modulators in plasma was detected by Milliplex 26-plex (Millipore Corp, St. Charles, Missouri, USA), according to the provided manufacturers’ protocol. This method was applied to analyze a broad range of immune modulators; IL-1β, IL-2, IL-4, IL-5, IL-6, IL-7, IL-8, IL-9, IL-10, IL-12, IL-13, IL-15, IL-17a,Tumor necrosis factor (TNF)-α, IFN-γ, monocyte chemoattractant protein (MCP)-1, interferon gamma-induced protein (IP)-10, macrophage inflammatory protein (MIP)-1α and β, eotaxin, RANTES, granulocyte colony-stimulating factor (G-CSF), granulocyte monocyte colony-stimulating factor (GM-CSF), basic fibroblast growth factor (b-FGF), vascular endothelial growth factor (VEGF), and platelet-derived growth factor (PDGF), with different roles and functionality.

### RNA isolation from plasma

Total RNA was isolated from plasma with the miRCURY™ RNA isolation kit Biofluids, (EXIQON, United States). This is a method for purification of RNAs smaller than 1000 nt (such as miRNAs) from biofluids like plasma. Samples were then analyzed according to the manufacturers’ protocol.

Due to a very low level of RNA in plasma, determination of the exact concentration is not possible by spectrophotometric reading. Therefore we used the biofluid input amount in PCR reaction as a measure as recommended by the manufacturer.

### Profiling and validation of microRNA expression by quantitative reverse transcription polymerase chain reaction (RT-qPCR)

Exiqon miRCURY Ready-to-Use PCR Human panel I V1.M (Exiqon miRNA qPCR panel) was used to identify the potentially affected plasma microRNAs following treatment. Five randomly selected patients, treated with 2 μg paricalcitol, were included in the initial profiling phase (pilot study). In the profiling step, out of 305 assays included in miRCURY LNA™ Universal RT miRNA PCR panel I, 290 assays were detected in at least one sample. The normalization was performed based on the average of the assays detected in all samples and for the present study, 97 assays were considered. The stability of the average of 97 microRNAs was higher than any single microRNA in the whole data set, as measured by the NormFinder software. The formula used to calculate the normalized Cq values was: Normalized Cq = average Cq (*n* = 10) – assay Cq (sample). A higher value thus indicated that the microRNA was more abundant in the particular sample. RNA spike-in for quality control of the RNA isolation and cDNA synthesis has been applied. RNA isolation control (UniSp2, UniSp4, UniSp5) were added to the purification to detect any differences in extraction efficiency. The cDNA synthesis control (UniSp6) was added in the reverse transcription reaction, giving the opportunity to evaluate the RT reaction. In addition to this a DNA spike-in (UniSp3) was present in all samples. Controls, no template control (NTC) and RNA spike-in, indicated good technical performance of the profiling experiment. The unsupervised analysis of the samples clustered robustly according to the groups (before vs. after treatment), and the treatment status did not contribute to the overall variation in the microRNA profiles. Identified miRs from the initial screening, were then subjected to validation.

In validation groups, patient and placebo samples (before and after vitamin D treatment), were used for miR analysis. Using the Universal cDNA synthesis kit II (miRCURY LNA™ Universal RT microRNA PCR, EXIQON, United States), 2 μl of template RNA was reverse transcribed to cDNA in a total volume of 10 μl according to the manufacturer’s recommendations. For RT-qPCR, ExiLENT SYBR Green master mix kit (miRCURY LNA™ Universal RT microRNA PCR, EXIQON, United States) was applied. 4 μl cDNA, PCR master mix, PCR primers, and nuclease-free water, were used for PCR reaction. Each PCR reaction was performed in duplicate with MicroAmp™ optical 384-well reaction plates using an ABI 7900 (Life Technology).

### Statistical analysis

Statistical analysis was made in GraphPad Prism 5, STATISTICA version 10 (Stat Soft, Inc., USA) and SPSS version 22 (IMB, 2011). For normally distributed lab data, we compared the three groups, before and after treatment, using one way ANOVA and repeated measures ANOVA. The pre-treatment levels of cytokines were compared between the three groups, using nonparametric Kruskal-Wallis. The post-treatment levels of cytokines were compared to the pre-treatment levels, within each group, using nonparametric Wilcoxon matched-pairs signed rank test, since values were not normally distributed. A *p* < 0.05 was considered significant. Line graphs were prepared by GraphPad Prism 5. To compare treated groups with the placebo group in terms of miRs, the change in expression of miRs (post minus pre-treatment or delta values) was used. Due to a non-normally distributed data, comparison between the three groups was performed by nonparametric Kruskal-Wallis. Scatter plots represented 25–75% interquartile range with a line at the median. A *p* < 0.05 was considered significant.

## Results

Thirty five out of 36 patients completed the trial. One individual perceived dizziness from the study drug (for that subject it was placebo) and declined to complete the intervention and to undergo the final measurements. There were no major adverse events or hospitalizations during the study.

### Baseline measurements

The study subjects, who were randomly assigned to three groups, were comparable in terms of sex (*p* = 0.47) but those in the placebo group were slightly older than those in the treated groups (*p* = 0.02). The prevalence of previous myocardial infarction was lower in the paricalcitol 2 μg-treated group. There was also a tendency to a higher number of patients with polycystic kidney disease in the 2 μg-treated group. The three groups were well matched in terms of eGFR, calcium, phosphate, 25-OH-vitamin D3 and PTH levels at baseline.

### Post treatment laboratory findings

The three groups did not differ in eGFR, calcium, phosphate, and 25-OH-vitamin D3 levels after treatment. There was a dose-dependent response in PTH levels. These data together with the clinical data have been explained thoroughly elsewhere [17]. There were no significant differences between the groups with regard to the total leukocyte, erythrocyte and platelet counts, as well as CRP and plasma albumin levels.

### Characterization of cytokines and chemokines in plasma

The pre-treatment measurements were well matched between placebo and treatment groups (1 and 2 μg paricalcitol).

VEGF and PDGF showed significantly decreased levels after treatment with 1 μg and 2 μg paricalcitol, so did IP-10 after treatment with 2 μg paricalcitol; as shown in Table 2. There were no changes in cytokine concentrations comparing pre- and post-measurements in the placebo group.

**Table 2:** The concentration of cytokines (pg/ml) in plasma, pre- and post-treatment within each group; placebo, 1 μg and 2 μg paricalcitol

Cytokine	Placebo Pre / ﻿﻿Post	*p*-value	Paricalcitol 1 μg Pre / Post	*p*-value	Paricalcitol 2 μg Pre / Post	*p*-value
VEGF^a^	20 (9–36) / 18 (13–21)	0.2	20 (15–31) / ﻿10 (3–15)	**0.01**	26 (9–60) / 10 (7–32)	**0.02**
PDGF^b^	1220 (719–3740) / 837 (411–3084)	0.2	1297 (960–1933) / 784 (561–915)	**0.005**	2811 (1447–3706) / 1234 (1003–2239)	**0.009**
IP-10^c^	342 (169–5167) / 332 (71–5091)	0.8	423 (353–2805) / 402 (274–614)	0.08	2628 (511–6200) / 492.5 (270–2022)	**0.02**

Values are given as median and interquartile range (25–75%). The analysis was done by nonparametric Wilcoxon matched-pairs signed rank test

The significant *p*-values are presented in bold

^a^Vascular endothelial growth factor

^b^Platelet-derived growth factor

^c^Interferon gamma-induced protein-10

We did not find any significant changes in the levels of other cytokines analyzed following treatment.

### Characterization of microRNAs in plasma

**Fig. 1:**
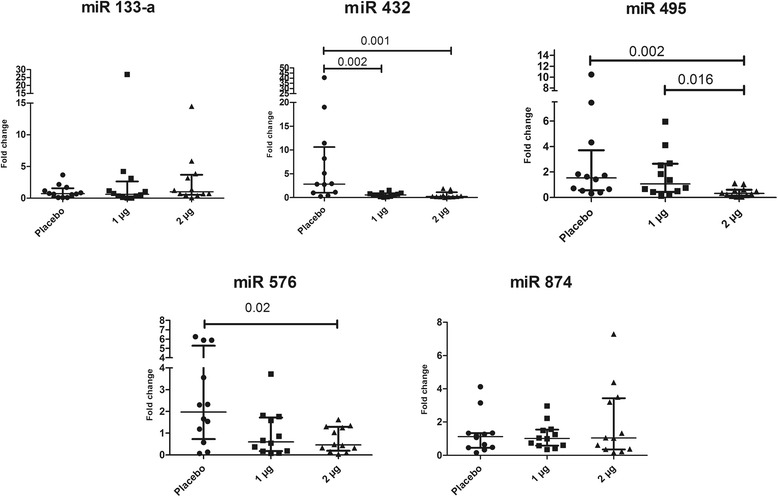
Significantly changed plasma microRNAs following treatment, comparing the three groups; placebo, 1 μg and 2 μg. Scatter plots represent 25–75% interquartile range with a line at the median

Based on the results from the pilot study (*p*-value and fold change), the five top-ranked down regulated microRNAs after VDRA treatment, were validated in all patients (Table 3). These five miRs were detected in plasma from both placebo and paricalcitol-treated patients. Following treatment with 2 μg of paricalcitol, miR 432 -5p, miR 495-3p, and miR 576-5p were significantly decreased (Fig. 1). A dose–response effect was also observed in the levels of miR 495.

**Table 3:** The top-ranked changed plasma microRNAs, after treatment compared to before treatment, in patients treated with 2 μg paricalcitol (Pilot study)

miR	*p*-value	Fold change
hsa-miR-576-5p	0.04	−1,92
hsa-miR-432-5p	0.05	−2,00
hsa-miR-133a-3p	0.05	−2,13
hsa-miR-495-3p	0.06	−2,07
hsa-miR-874-3p	0.07	−1,94

Five patients were randomly selected to undergo the pilot analysis. The changed miRs were ranked according to the *p*-value and fold change. The five top-ranked miRs were chosen for further validation

There was no significant statistical correlation between the clinical data (measurements of endothelial function and microcirculation) and the cytokine and miR measurements.

## Discussion

In the present study we show that 12 weeks of treatment with paricalcitol, an active synthetic vitamin D analogue, in CKD patients reduces pro-inflammatory cytokines, and microRNAs involved in the regulation of atherosclerosis, immune response and platelet function.

End stage renal disease (ESRD) patients have high plasma levels of inflammatory cytokines [5, 8]. This pro-inflammatory state affects vascular endothelial cells and is supposed to induce atherosclerosis and angiogenesis [22]. In the present study we observed a lower level of PDGF and VEGF, following treatment with paricalcitol, which is noteworthy since PDGF and VEGF particularly promote angiogenesis as well as progression of atherosclerotic plaques [23, 24]. Moreover, IP-10 (CXCL10), which attracts immune cells to the site of inflammation and maintains the inflammatory process [25, 26], decreased significantly with the higher dose of paricalcitol.

Development of CVD in CKD patients is partly due to endothelial dysfunction and a chronic inflammatory state [3]. Vitamin D is of great interest as a hormone involved in many physiological processes in the body such as regulation of immune responses, fibrosis and endothelial function [20, 27]. Vitamin D deficiency is also strongly associated with an increased risk of cardiovascular events [13]. In CKD patients, treatment with the active forms of vitamin D has been shown to reduce albuminuria, and in some trials an associated reduction in blood pressure [14, 28]. Cardiovascular end-point studies in patients with moderate CKD (stage 3–4) are missing, however four interventional studies show positive effects of vitamin D treatment on endothelial function, an independent risk factor for cardiovascular diseases [16–19].

Several studies have indicated an association between vitamin D levels and inflammation [29], however there are some conflicting data regarding the impact of vitamin D and its analogues on pro-inflammatory cytokines and endothelial dysfunction [30, 31]. In a trial with hemodialysis patients, plasma levels of TNFα, IL6 as well as their mRNA expression in peripheral blood mononuclear cells (PBMCs) decreased significantly following treatment with paricalcitol for 12 weeks [30]. Our study supports the notion that vitamin D deficiency plays a role in the activated inflammatory state and endothelial dysfunction seen in CKD patients.

In recent years, circulating miRs have drawn attention as a new class of biomarkers and are thought to regulate the process of inflammation and fibrosis in CKD and cardiovascular diseases [32, 33]. We showed a down-regulation of miR 432 in plasma, following treatment with paricalcitol. Atherosclerotic lesions comprise low levels of DNA methylation, which have been proposed to lead to activation of a gene cluster with several miRs, among them miR 432 [34]. It has also been shown that low levels of miR 432 leads to activation of the Wnt-β-catenin pathway [35], which in turn dampens the NFkB activity and production of inflammatory cytokines [36, 37]. Thus, a lower level of miR 432 is likely to suggest a decline in inflammatory responses.

In the present study, paricalcitol treatment also reduced the levels of miR 495. It is known that miR 495 plays a role in regulating the function and reactivity of platelets [38]. In a mouse study, inhibition of a class of miRs including miR 495, led to an improved vascular and blood flow recovery following ischemia [39]. We also found that paricalcitol reduced levels of miR 576. The role of miR 576 is not clear, but it appears to be involved in the pro-inflammatory immune response since over expression of miR 576 has been shown in patients with the infectious disease pertussis [40].

Our major limitation of the present study is the small sample size. We therefore were not able to investigate either the potential effects of varying doses of paricalcitol, nor gender, on the miRNAs or cytokine levels. Initially we aimed to correlate the clinical data on endothelial function with the cytokine and miR findings, but due to the limited sample size this was not feasible. Even so, the findings in this randomized trial indicate that the treatment impact on pro-inflammatory and pro-atherosclerotic molecules may contribute to previously reported effects on endothelial function in CKD patients.

## Conclusion

We show that treatment with an active vitamin D analogue in moderate CKD reduces pro-inflammatory cytokines and down regulates miRs involved in atherosclerosis, immune response and platelet function. Our results support the hypothesis that activated vitamin D treatment dampens the inflammatory state in moderate CKD patients, which in turn affects endothelial and vascular function. The potentially protective role of vitamin D receptor activation in the inflammatory processes regarding long-term outcomes in CKD patients warrants further studies.

## Abbreviations

ACE-I: Angiotensin converting enzyme inhibitor
ARB: Angiotensin receptor blocker
b-FGF: Basic fibroblast growth factor
CKD: Chronic kidney disease
CVD: Cardiovascular diseases
eGFR: Estimated glomerular filtration rate
ESRD: End stage renal disease
GM-CSF: Granulocyte monocyte colony-stimulating factor
IP-10: Interferon gamma-induced protein 10
MCP: Monocyte chemoattractant protein
MIP: Macrophage inflammatory protein
miRs: Micro RNAs
NTC: No template control
PBMCs: Peripheral blood mononuclear cells
PDGF: Platelet-derived growth factor
RAAS: Renin-angiotensin-aldosterone system
TNF: Tumor necrosis factor
VDRA: Vitamin D receptor activator
VEGF: Vascular endothelial growth factor
